# Small Antennas for Wearable Sensor Networks: Impact of the Electromagnetic Properties of the Textiles on Antenna Performance

**DOI:** 10.3390/s20185157

**Published:** 2020-09-10

**Authors:** Gabriela Atanasova, Nikolay Atanasov

**Affiliations:** 1 Department of Communication and Computer Engineering, South-West University “Neofit Rilski”, 2700 Blagoevgrad, Bulgaria; natanasov@swu.bg; 2 Electromagnetic Compatibility Laboratory, Bulgarian Institute of Metrology, 1040 Sofia, Bulgaria

**Keywords:** small antenna, textile antenna, wearable antenna, SAR, flexible antenna, low-profile antenna, sensor network, active test

## Abstract

The rapid development of wearable wireless sensor networks (W-WSNs) has created high demand for small and flexible antennas. In this paper, we present small, flexible, low-profile, light-weight all-textile antennas for application in W-WSNs and investigate the impact of the textile materials on the antenna performance. A step-by-step procedure for design, fabrication and measurement of small wearable backed antennas for application in W-WSNs is also suggested. Based on the procedure, an antenna on a denim substrate is designed as a benchmark. It demonstrates very small dimensions and a low-profile, all while achieving a bandwidth (|S_11_| < −6 dB) of 285 MHz from 2.266 to 2.551 GHz, radiation efficiency more than 12% in free space and more than 6% on the phantom. Also, the peak 10 g average SAR is 0.15 W/kg. The performance of the prototype of the proposed antenna was also evaluated using an active test. To investigate the impact of the textile materials on the antenna performance, the antenna geometry was studied on cotton, polyamide-elastane and polyester substrates. It has been observed that the lower the loss tangent of the substrate material, the narrower the bandwidth. Moreover, the higher the loss tangent of the substrate, the lower the radiation efficiency and SAR.

## 1. Introduction

Wearable wireless sensor networks (W-WSNs) can be applied in diverse areas, including health care (clinical diagnostics, rehabilitation), sports (athlete activity profile, energy expenditure during training) and work safety (monitors for the temperature, humidity, CO_2_) [1,2]. These networks are a particular case where sensors are deployed on the user clothing and/or directly on the body to measure physiological signals of a human and/or to monitor its environment [3,4,5]. Hence, each W-WSN consists of multiple wearable sensor nodes which are capable of communicating with each other (on-body communications) or with external devices (off-body communications) allowing a connection with a monitoring centre (smartphone, local or cloud webserver). Several frequency bands (such as 2.36–2.38 GHz medical body area network (MBAN), 2.4–2.48 GHz and 5.75–5.82 GHz industrial, scientific, and medical (ISM) bands) and wireless technologies such as IEEE 802.11, IEEE 802.15, LTE, LoRA, etc. are used to connect wearable sensor nodes. These wireless technologies require each wearable sensor node to be equipped with a sensing element, processor, memory, power module, transceiver and an antenna [2,4,6].

The antenna plays a key role in the link performance of each wearable wireless sensor node because it determines the reliability of the wireless link and directly influences the power consumption of the node [2]. Moreover, small, flexible, low-profile, and light-weight wearable antennas based on materials which are deformable, twistable and stretchable are needed because the sensor node needs to be seamlessly worn [7]. Most of the proposed flexible wearable antennas are based on polymers [5,8,9], textiles [1,7,10,11,12,13,14,15,16,17] or flexible ceramics [18].

Among flexible materials, textiles are the most widely employed materials for wearable antennas due to their ease of integration on the clothes. The operating frequency bands and radiation efficiency of such a kind of antenna structure can be controlled by proper selection of the antenna topology, substrate thickness and substrate electromagnetic (EM) properties [1,19,20]. Consequently, design of the all-textile wearable antennas requires precise knowledge of the EM properties of textiles used for the antenna substrate at the frequencies of interest.

The second major challenge when designing wearable antennas is the performance reduction (such as shifting of the resonant frequency, changing of the input impedance, reducing the radiation efficiency of the antenna) caused by the specific environment, in which wearable antennas operate (close to the human body) [5,17,21,22]. Hence, antenna topologies with high body-antenna isolation are needed to guarantee a satisfactory performance in varying operating conditions and to reduce the specific absorption rate (SAR) [2].

In the literature, a diverse range of techniques have been reported for reducing interaction between the antenna and human tissue. One popular technique to reduce electromagnetic coupling between the antenna and human body is to use metamaterials such as electromagnetic bandgap [10,11,12,13,14] and artificial magnetic conducting surfaces [16,17]. Another technique is to use a reflector [1,5,8,15] or a full ground plane [7].

Also an essential aspect would be considered when designing antennas for wearable sensor nodes is miniaturization. Most of the proposed antenna designs still suffer from relatively large size [1,9,14,15,16,17] or high profile [5,10,11,12] and do not meet the requirements (for low-profile and small size) of the antennas for wearable wireless sensor nodes. Consequently, the design of antennas for wearable wireless sensor nodes is a complex task. Generally, in wearable antenna design, electrical, mechanical, and safety requirements described in [2] should be taken into account.

In this paper, we extend our conference paper [1] where the characterization of the EM properties of the regular textiles and study of their effects on performance (radiation efficiency, reflection coefficient magnitude, bandwidth, and maximum gain) of wearable backed antennas have been presented. Now, details about the design, manufacturing and performance measurements of small wearable backed antennas for applications in sensor nodes are provided. The proposed step-by-step methodology allows us to design and experimentally realize new small, low-profile, lightweight and flexible all-textile antennas with high body-antenna isolation. Compared with [1], additional contents of this expansion paper are as follows: (1) a step-by-step design, fabrication and measurement procedure of small wearable backed antennas for application in sensor nodes; (2) new small all-textile antennas for potential integration into everyday clothing; (3) a study (by simulations and measurements) of the antenna performance in free space and on a phantom of the human body.

## 2. Design, Fabrication and Measurement of Small Antennas for W-WSNs

**Figure 1:**
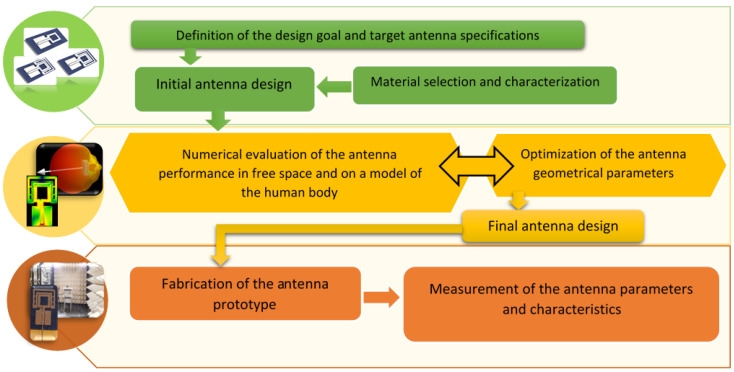
Block diagram of the design, optimization, fabrication and measurement process of small antennas for W-WSNs.

Figure 1 shows the generic flowchart of the design, optimization, fabrication and measurement processes of small antennas for W-WSNs used in this work. As shown in Figure 1, the antenna design process starts with the definition of the design goal and target antenna specifications. The design goal of this work is to create new small low-profile all-textile antennas which provide sufficient radiation efficiency and appropriate isolation to the human body for off-body communications in W-WSNs, for potential integration into everyday clothing. The target antenna specifications and required performance associated with this goal are set out below in Table 1.

The frequency range pointed in Table 1 is chosen because most of the devices for off-body communications are designed for operation in the unregulated 2.36–2.38 GHz MBAN and 2.4–2.48 ISM frequency bands [3,5,6,10,11,22]. The minimum value of the radiation efficiency pointed in Table 1 is chosen based on a survey of radiation efficiency of internal antennas in real mobile phones [23]. The results from the measurements of the efficiency of the mobile-phone antennas show that average handset radiation efficiency varies between 4.5% and 20%. As expected, the antenna with the smallest size (antenna had a maximum length of 36 mm) has the lowest radiation efficiency (4.5%).

### 2.1. Initial Antenna Design

The initial design of the antenna starts with the choice of an antenna geometry and a material for the substrate. There is no single specific type of geometry for wearable antennas. The most prevalent types are dipole [5,8], monopole [1,10,12,14,15,16,17] and patch antennas [7,9,11]. The choice of geometry needs to be guided from the design requirements such as simple structure, small size, low-profile and light-weight. Consequently, the antenna needs to be physically small.

In this work, an antenna geometry based on a loop antenna was chosen. This structure is one of the simplest small radiators and provides good matching with many types of feedings, such as coaxial cables and planar transmission lines.

Another point to keep in mind during the initial design is the choice of materials for the antenna’s elements and characterization of their electromagnetic properties, as shown in Figure 1. When designing antennas for potential integration into everyday clothing, the substrate material is predetermined. This is the textile from which the garment is made and in which the antenna will be embedded. Measurements of the complex permittivity of the chosen textile can be carried out using the resonant or non-resonant methods [1]. Conductive fabrics or threads are the most widely used materials for radiating elements. The information about dc conductivity (or sheet resistance) of the conductive textiles usually is available from the datasheets provided by the manufacturers.

In this study, conventional fabrics with natural fibre (denim and cotton), as well as synthetic fibre (polyester and polyamide-elastane) have been selected as substrates to develop small, low-profile, light-weight all-textile antennas. Conductive fabric has been chosen as a material for the radiating elements and reflector. More details about the EM properties of the selected textiles can be found in our conference paper [1].

**Figure 2:**
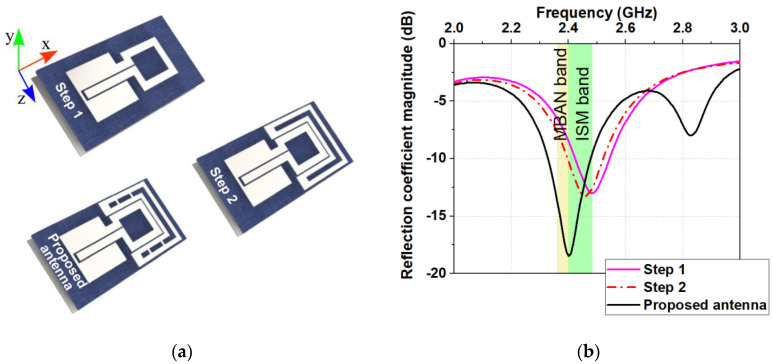
Antenna geometry: (**a**) Design steps; (**b**) Simulated reflection coefficient magnitudes versus frequency.

The steps to design a small all-textile antenna that meets the specifications presented in Table 1 are depicted in Figure 2. Figure 2a depicts the structure of the proposed antenna in the first step of the design process. It is composed of a rectangular loop, coplanar waveguide (CPW) transmission line, substrate, and a reflector. The choice of the CPW to feed the antenna was because it offers a single-layer manufacturing process. The substrate is denim with a real part of relative permittivity ($\varepsilon_{r}^{\prime }$) 1.878, and a loss tangent (tan δ) 0.0594 at 2.565 GHz. The thickness of the substrate is 1.5 mm (comprised of three-layer denim) with density from 1.54 g/cm^3^ [1,2]. This substrate thickness enables us to obtain a small antenna with a low-profile. The reflector was implemented in the antenna’s structure to reduce the effect of the human body over the antenna performance and SAR. It was chosen due to its simple form. Also, it is relatively easy for numerical modelling and manufacturing. The geometrical dimensions (length and width) of the loop strips and CPW were tuned to yield a resonance at around 2.47 GHz. Figure 2b shows the reflection coefficient magnitude of the proposed antenna during the design process. In the next step, an arc-shaped parasitic element was added at a close distance to the loop structure (see Figure 2a, Step 2). It is observed that the resonant frequency shifts to a lower frequency while the bandwidth and the impedance matching of the antenna are not changed. Finally, to tune the input resistance of the proposed antenna closer to 50 Ohms and broader bandwidth, four parasitic elements were inserted in the arc-shaped loop (see Figure 2a). This enables a good impedance match with a lower than −6 dB bandwidth (from 2.266 to 2.551 GHz) of approximately 285 MHz and a resonant frequency of 2.4 GHz, as shown in Figure 2b.

### 2.2. Numerical Evaluation of the Antenna Performance in Free Space and on a Model of the Human Body

Design, optimization and numerical evaluation of the antenna performance are carried out using the full-wave electromagnetic commercial software Remcom xFDTD (xFDTD, Remcom Inc., State College, PA, USA). This EM software packet is based on the finite difference time domain (FDTD) method which is extensively used for modelling antenna structures and human body models.

The following parameters are set in each simulation in order to obtain more accurate results. First, FDTD cell size of 0.5 mm × 0.5 mm × 0.5 mm is used for the antenna geometry. For the human body model and rest of the space, an inhomogeneous mesh having an increasing cell size from 0.5 to 1 mm is applied. All calculations continue until steady-state is reached. For the analyzed small antenna with a denim substrate, the steady-state is observed after 10,000 time-steps. A 7-layer perfect matched layer absorbing boundary condition is used.

Since the designed antenna is expected to be in close proximity or mounted directly on the different parts of the human body during the real operating conditions in a W-WSN, the design and optimization were made first in the free space. After that, geometrical dimensions of the antenna structure were optimized on a human body model. A homogeneous numerical model with dimensions 180 mm, 120 mm and 150 mm was employed to mimic the human body ($\varepsilon_{r}^{\prime }\;$= 40.805, σ = 2.33 S/m, and ρ = 1166 kg/m^3^). The selected human model dimensions are those for the flat phantom pointed in standards EN 62209-2:2010 and IEEE Std.1528-2013 for the fixed frequency of 2.45 GHz so that the effect of the power reflection at the model surface on the peak 10 g average SAR is negligible (less than 1%).

**Figure 3:**
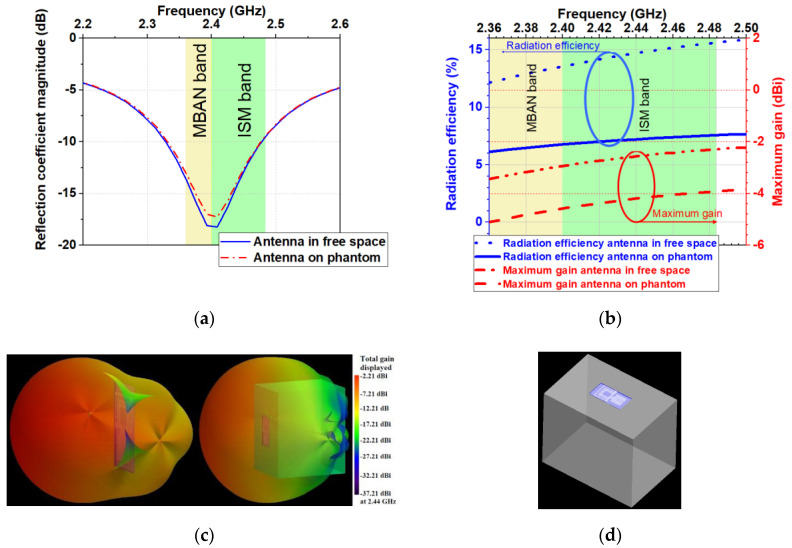
Simulated: (**a**) Reflection coefficient magnitudes versus frequency; (**b**) Radiation efficiency and maximum gain versus frequency; (**c**) 3D radiation patterns of the antenna at 2.44 GHz in the free space and placed on the surface of the phantom; (**d**) Model of the antenna and phantom.

The antenna was positioned on the surface of the phantom (the distance between the antenna and the phantom is 0 mm, as shown in Figure 3d) to study the effect of the human body on the antenna performance and SAR in the worst-case scenario.

The free space and on-phantom reflection coefficient magnitudes (|S_11_|) of the optimized antenna are presented in Figure 3a. In the free space, the antenna bandwidth (|S_11_|<−6 dB) is 285 MHz. As we can see the homogeneous phantom does not cause detuning effects on the resonant frequency. The |S_11_| curve and operating bandwidth for the case where the antenna is mounted on the phantom are the same as the case in free space.

The total radiation efficiency and maximum gain of the optimized antenna in the free space and on the phantom at the frequency bands of interest are displayed in Figure 3b. As we can see, the phantom causes a reduction in total radiation efficiency and maximum gain. The total radiation efficiency and maximum gain are decreased by a factor of about 0.5 when the antenna is placed on the phantom. Also, the total radiation efficiency and maximum gain show an increasing trend with increasing the frequency. Figure 3b shows that across the bandwidth of 2.36–2.5 GHz the simulated radiation efficiency varies from 12% to 16% when the antenna is in the free space and from 6% to 7.8% when it is placed on the phantom.

From Figure 3c we can see that the antenna exhibits unidirectional radiation pattern. The front-to-back ratio is 12.20 dB in the free space and 22.55 dB on the phantom. Another essential question to be considered in designing antennas for W-WSNs is about concerning health hazard. Hence, to address this question, a more thorough evaluation and characterization of the SAR in the human body model were carried out. According to the safety guidelines and standard [24,25], the obtained peak 1 g and 10 g average SAR values should not exceed 1.6 W/kg and 2 W/kg.

**Figure 4:**
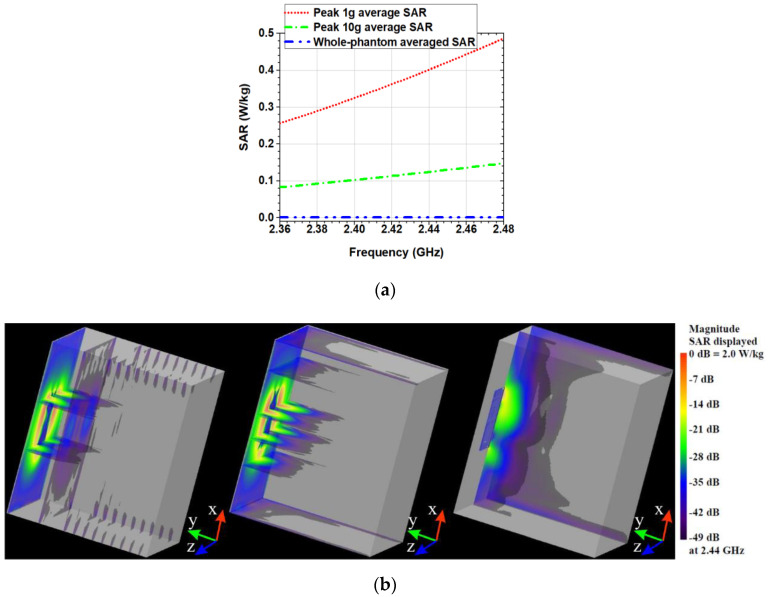
FDTD-computed SAR: (**a**) peak 1 g and 10 g average SAR, and whole-phantom averaged SAR versus frequency; (**b**) Distributions in xy-, yz- and zx-plane at 2.44 GHz and scale.

Figure 4a shows the peak 1 g and 10 g FDTD-computed average SAR generated from the antenna in the phantom, in the MBAN and ISM bands when the antenna is positioned on the surface of the phantom (the distance between the antenna and the phantom is 0 mm). The results were normalized to a net input power of 100 mW. It can be seen that the peak SAR is frequency dependent. In general, the SAR increases as the frequency increases. For the considered input power, the peak 1 g and 10 g average SAR produced in the phantom are 0.533 and 0.148 W/kg, respectively at 2.48 GHz. The peak 10 g average SAR values are much lower than the maximum allowed value of 2 W/kg as required by the ICNIRP [24]. Moreover, the peak 10 g average SAR is found to be equal to ~2 W/kg when the net input power for the proposed antenna is 1353 mW. That is, the net input power as high as 1353 mW guarantee conformance with the safety guidelines imposed by the ICNIRP [24].

From the results presented in the Figure 4, it can be concluded that the proposed antenna exhibits low SAR values (peak 10 g average SAR is about ten times lower than the maximum allowed value of 2 W/kg) due to the shielding effect of the reflector and also due to the unidirectional radiation pattern of the antenna.

The SAR distributions on the surface and inside the phantom in the xy-, yz- and zx-planes are shown in Figure 4b. In the figure, we observe that the SAR values around the antenna edges are higher.

Also, under real-case scenarios, the distance between the wearable antenna and the human body may be changed, which may lead to a change in the antenna performance and SAR. Hence, numerical simulations were performed at distances 0 mm, 5 mm and 10 mm between the antenna and the human body model in order to investigate the robustness of the proposed design to effects of the human body loading.

**Figure 5:**
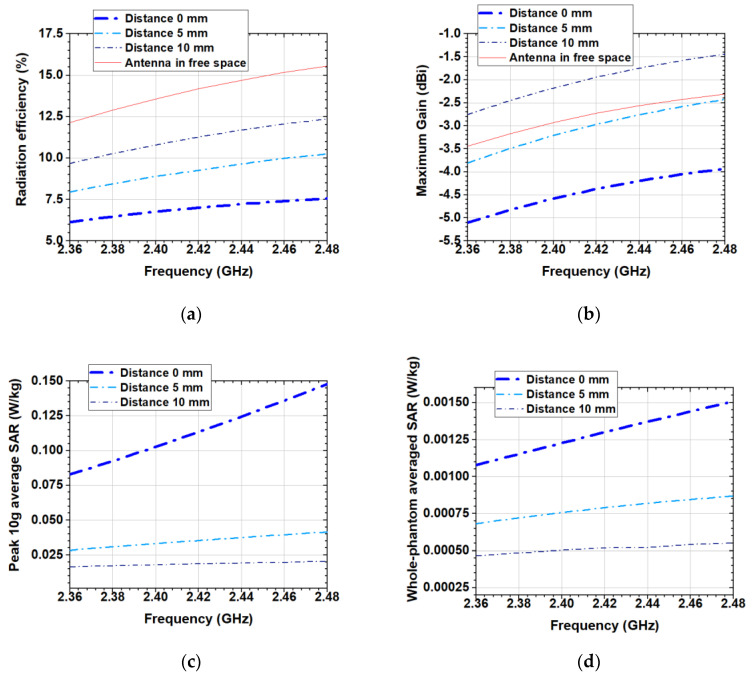
Simulated: (**a**) Radiation efficiency; (**b**) Maximum gain; (**c**) peak 10 g average SAR; (**d**) whole-phantom averaged SAR at distances 0 mm, 5 mm and 10 mm between the antenna and the human body model.

Figure 5 presents the variation of the antenna radiation efficiency, maximum gain, peak 10 g average SAR and whole-phantom averaged SAR (at 100 mW net input power) versus the frequency for the distances 0 mm, 5 mm and 10 mm between the antenna and the phantom. As might be expected, the radiation efficiency increases with distance, while the peak 10 g average SAR and whole-phantom averaged SAR decrease. Also, the maximum gain shows an increasing trend with increasing the distance between the antenna and the phantom. From Figure 5b we see that the gain at a distance 10 mm from the phantom is larger than that in the free space. This is because the phantom, in this case, acts as a reflector, which enhances radiation in the direction opposed to the human body model.

Moreover, when the designed antenna is a part of a wearable wireless sensor node intended for a medical purpose such as tracking, recording, and monitoring of biomedical signals (used in a medical or home healthcare environment), it is essential to create an electromagnetically compatible device to minimize interference with other devices. In this case, the wearable wireless sensor node has to be designed and validated as a medical device (or medical electrical equipment according to the definition in IEC 60601-1). The standards which specify tests and requirements for the electromagnetic compatibility of the medical electrical equipment are IEC 60601-1-2 and IEC 60601-1-11.

Finally, examining the results from numerical simulation presented in Figure 3, Figure 4 and Figure 5 as compared to the specifications of Table 1 illustrates that, the proposed antenna satisfies the requirements for application in W-WSNs.

### 2.3. Fabrication of the Antenna Prototype

For the fabrication of the radiating elements of a textile antenna, it is possible to use the inkjet printing [26], embroidery [27] or cut-transfer-glueing process [2]. The choice usually is made on the base of the materials used for the radiating elements.

**Figure 6:**
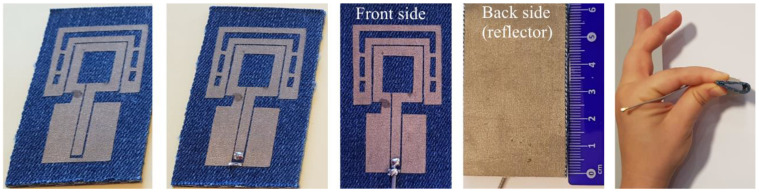
Photographs of the antenna’s prototype during the fabrication process.

To fabricate the antenna’s prototype, we used the cut-transfer-glueing process. By using the cutting machine, the antenna’s elements were cut into the designed shapes with high accuracy (a tolerance of ±0.01 mm). A highly conductive fabric (DC conductivity 2.5 × 10^5^ S/m and 0.08 mm of thickness) was used for the conductive elements of the antenna. Next, both radiating elements and reflector were attached to the denim substrate by using a polymer tape that is activated by ironing. A coaxial cable (diameter 1.13 mm and length of 200 mm) with a 50 Ω U.FL connector was soldered to the CPW using a low-temperature solder wire. First, the soldering points of the coaxial cable with the conductive fabric were tin-plated at 250 °C (heating time 2.5 s), as shown in Figure 6. Then, the coaxial cable inner conductor was soldered on top of the middle conductor of the CPW while the coaxial cable braid shield was soldered to the CPW ground plane.

### 2.4. Measurements

The performance of the prototype of the designed antenna may be measured by using passive and active tests. In the passive tests, the prototype is connected to the measuring equipment (a network analyzer, signal generator, receiver, or spectrum analyzer) using a coaxial cable. In these tests, the antenna reflection coefficient magnitude (|S_11_|), bandwidth, gain, radiation pattern and radiation efficiency are measured. The detailed definition of the antenna parameters and their measurement methods are presented in [2,28,29].

Moreover, a full verification of the antenna design requires more extensive tests, which represents the behaviour of the antenna under practical operating conditions, called active tests [2,29,30]. In these measurements, a network simulator (or a radio communication test module) is used to set up a connection to the antenna under test, that is connected to a sensor node to reproduce a real-world performance. In order to conduct accurate and repeatable measurements, the testing needs to perform inside a chamber (anechoic or reverberation) with a controlled environment [2,28].

For antennas used near or on the human body such as antennas intended for sensor nodes in W-WSNs experimental measurements of the antenna parameters and characteristics on a physical model of the human body (called phantom) are also required. There are three types of phantoms for experimental use: solid, semi-solid, and liquid [31]. SAR measurements can be made using a robotic system, associated test equipment and a liquid phantom or using an infrared camera, associated test equipment and a solid or semi-solid phantom.

**Figure 7:**
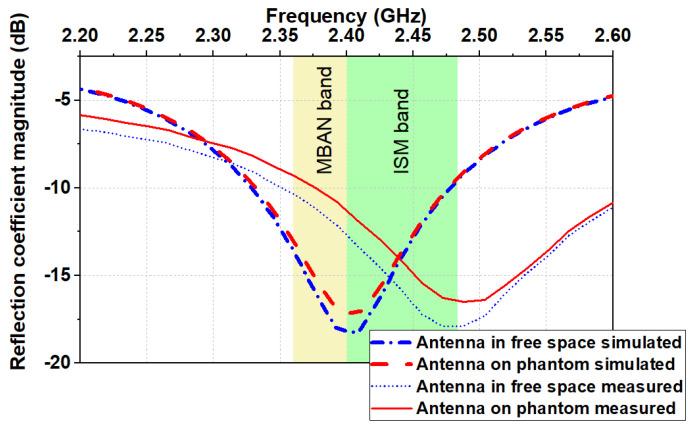
Simulated and measured reflection coefficient magnitudes versus frequency in the free space and on the semi-solid phantom.

In order to assess the performance of the proposed antenna, |S_11_| was evaluated when the antenna is placed on a semi-solid phantom and in the case of free space condition. The semi-solid phantom (dimensions 180 mm (length) × 120 mm (width) × 150 mm (depth)) was fabricated accordingly to the recipe and technique described in [31]. The measurement of |S_11_| were performed using a Tektronix TTR503A vector network analyzer. As shown in Figure 7, when the antenna is placed on the semi-solid phantom, the |S_11_| remains almost unchanged. By comparing simulated and measured results, we observe a good agreement between them with a slight shift in the resonant frequency.

In order to get a complete performance of the fabricated prototype, active tests were carried out. The first set of measurements was performed in a semi-anechoic chamber. Next, in order to take into account, the environmental variability, the measurements were repeated in a shielded room. All tests were carried out for both scenarios: (1) in the free space and (2) when the antenna is placed on the phantom.

**Figure 8:**
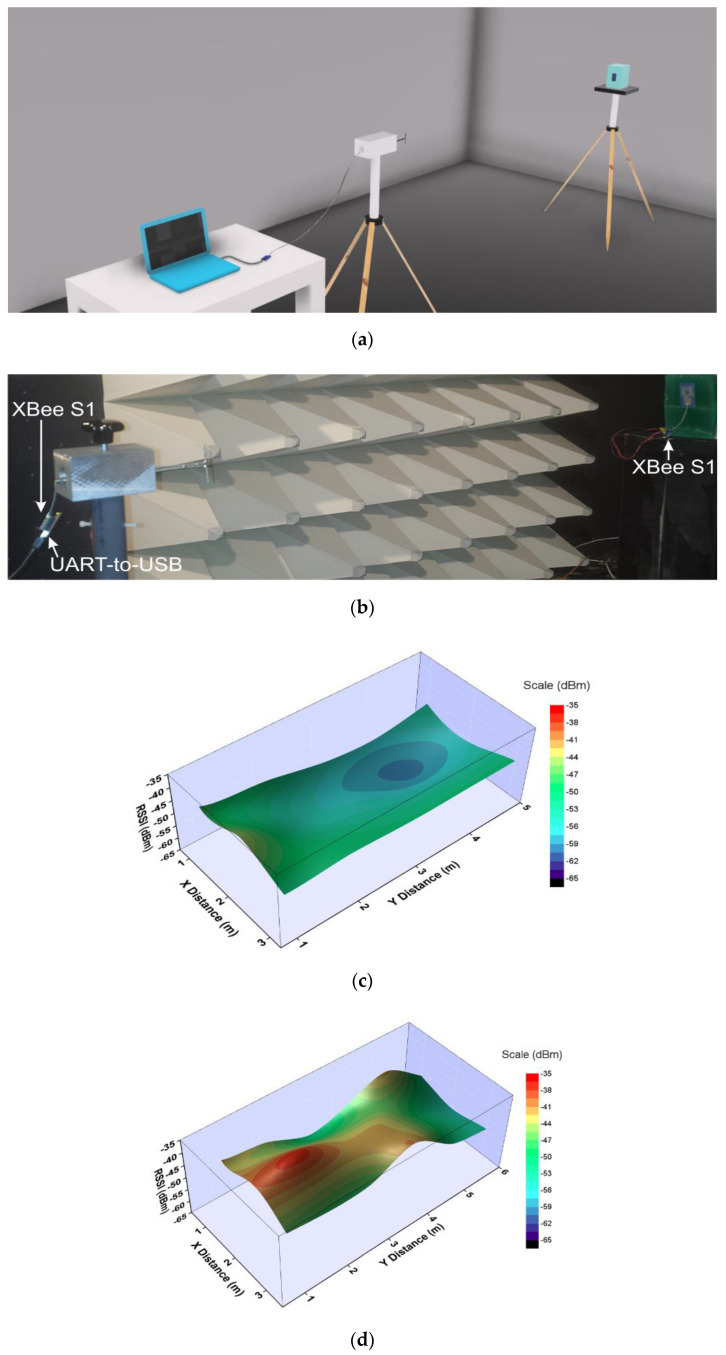

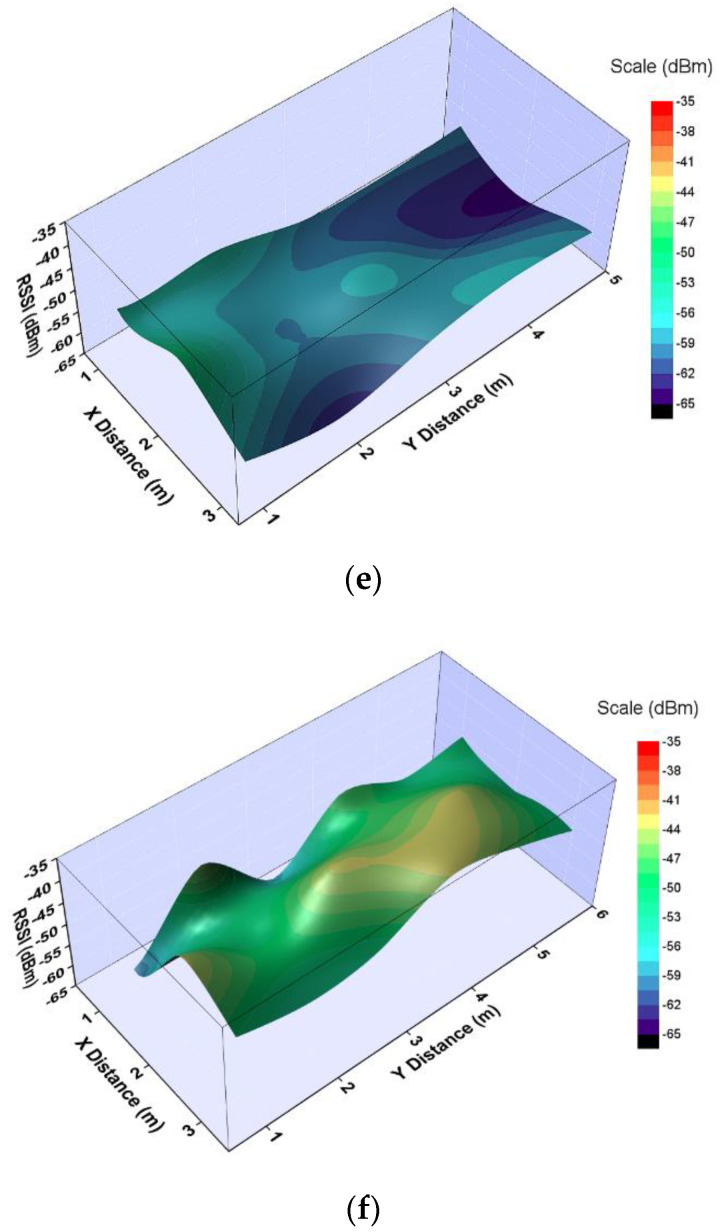
RSSI: (**a**) Simple drawing of the configuration of the test setup; (**b**) Photograph of the test setup in the semi-anechoic chamber; (**c**) Distribution in the semi-anechoic chamber in the free space; (**d**) Distribution in the shielded room in the free space; (**e**) Distribution in the semi-anechoic chamber when the antenna is on the semi-solid phantom; (**f**) Distribution in the shielded room when the antenna is on the semi-solid phantom.

Two XBee S1 (DiGi International, Hopkins, MN, USA) modules were used in all tests as wireless nodes. One of the nodes was connected by a coaxial cable with a dipole antenna that has a resonant frequency of 2.44 GHz. The dipole antenna was in a vertical orientation. Next, the wireless node was connected to a personal computer via a UART-to-USB controller (Figure 8a,b) and was configured to generate a continuously repeated pseudo-random signal of 100 packets (each packet contains 50 bytes). The second wireless node was connected to the fabricated antenna prototype. The wireless nodes were connected, running XCTU software. On the XCTU, the transmission power, operating frequency and data rate, were set to −1 dBm, 2.41 GHz, and 9.6 kbits/s, respectively.

The semi-anechoic chamber was divided into 15 specific positions (three columns and five rows). The dipole antenna connected to the XBee node was stationary. At the same time, the other XBee node connected to the antenna prototype was located in each of described 15 specific positions in line-of-sight to the dipole. Both antennas were placed at a high of 1.33 m.

A range test was performed in order to determine the range and link quality between the nodes representing a real-world scenario. During the range test, XCTU sends data packets from the stationary XBee node to the remote node and waits for the echo to be returned from the remote node to the stationary node. Also, during this test, the XCTU determines RSSI (Received Signal Strength Indicator) value and calculates the packet error rate.

The distributions over the xy-plane of the measured RSSI in the semi-anechoic chamber and shielded room are shown in Figure 8. We can see that in the free space the measured RSSI is between −44 and −69 dBm depending on the specific antenna position in the semi-anechoic chamber (see Figure 8c). Also, when the distance between the antennas increases, the measured RSSIs decrease. When moving from semi-anechoic to shielded room scenario, it can be seen that in the free space the measured RSSIs vary between −37 and −56 dBm. A positive interference is clearly seen in Figure 8d when the distance between the antennas is 2 m (the measured RSSI is −37 dBm). Moreover, when the distance between the antennas is 6 m, the measured RSSI decreases to −56 dBm. Fluctuations of the RSSI values in the shielded room are due to constructive and destructive interference.

When the antenna is placed on the phantom, the measured RSSIs were in the range of −50 to −64 dBm in the semi-anechoic chamber (see Figure 8e) and in the range of −44 to −60 dBm in the shielded room (Figure 8f). Comparing the results with the free space scenario, we can conclude that the measured RSSIs when the antenna is on the phantom are lower (with about 6 dB) than those in the free space. These differences in RSSIs are attributed to the reduction in the radiation efficiency and gain when the antenna is on the phantom. We also observed that at all measurements, the packet error rate was zero.

Comparing the results, we see that at the same positions, the measured RSSIs in the semi-anechoic chamber are lower than those in the shielded room. This is since the more of the reflections in the semi-anechoic chamber are eliminated while in the shielded room, the propagation occurs by multiple reflections in the environment, which results in an additional energy contribution.

From the results in Figure 8, it is possible to conclude that the proposed antenna shows very good RSSI values both in the semi-anechoic chamber and shielded room which satisfies well the requirement of the receiver sensitivity in W-WSNs (−94 dBm) [32].

## 3. Impact of EM Properties of the Textile Materials on the Performance of the Small Antennas for W-WSNs

### 3.1. Antenna Designs

This section investigates the effect of the EM properties of different textile materials on the performance of small low-profile backed antennas. Three antenna structures: (1) Antenna with a cotton substrate, (2) Antenna with a polyamide-elastane substrate and (3) Antenna with a polyester substrate were developed. Here, each antenna has a substrate thickness of 1.5 mm, with a real part of the relative permittivity of 1.6321 (cotton), 1.5493 (polyamide-elastane) and 1.6202 (polyester), respectively. The loss tangent of the textile substrates is 0.0439 (cotton), 0.0146 (polyamide-elastane) and 0.0051 (polyester). In the design of the antennas (with substrates from cotton, polyester and polyamide-elastane) the configuration of the antenna with denim substrate was used. The difference being that at each substrate, the geometrical dimensions of the loop and parasite elements were optimized for maximum radiation efficiency and optimal impedance matching in the desired bandwidth.

**Figure 9:**
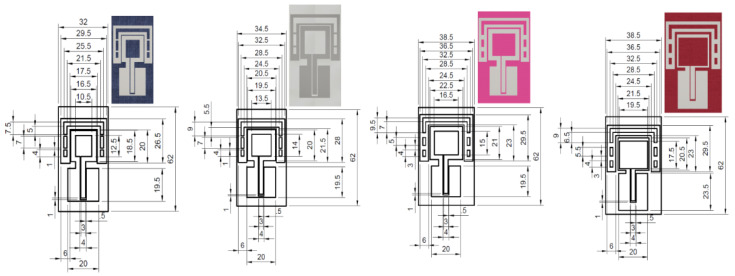
Configuration of the antennas with denim, cotton, polyamide-elastane and polyester substrates.

Figure 9 shows the structure and geometrical dimensions for each antenna. It is seen that in order to provide good impedance matching at the resonant frequency of 2.4 GHz, the perimeter of the square-loop on the denim substrate is 72 mm while the perimeter of the square-loop on the polyester substrate is 84 mm. Also, the loop strips width of the antenna with a polyester substrate are decreased to 1 mm to enhance the bandwidth.

From the results presented in the Figure 9, we can conclude, that the real part of the permittivity of the antenna substrate has consequences on the overall antenna size. As expected, with the increasing of the substrate material permittivity, the overall size of the antenna decreases [1,2,28].

A comparison with previously reported designs shows that the overall size of each of the four proposed antennas is between 25% and 90% smaller than the antennas in [1,9,10,12,14,15,26]. Moreover, the proposed antennas have a lower profile than [5,8,10,11,12,14].

### 3.2. Results and Discussion

Two scenarios were numerically studied when the antennas are: (1) in the free space and (2) placed on the semisolid phantom.

**Figure 10:**
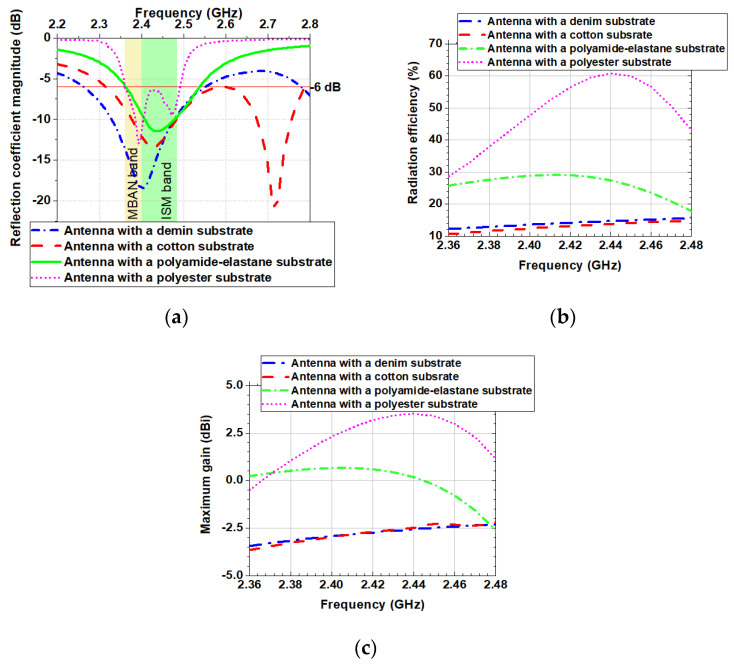
Simulated in the free space: (**a**) Reflection coefficient magnitudes versus frequency; (**b**) Radiation efficiency versus frequency; (**c**) Maximum gain versus frequency.

As shown in Figure 10a in free space, the antennas with denim and cotton substrates have a bandwidth (|S_11_| < −6 dB) of 285 and 269 MHz, respectively. In contrast, antennas with substrates from polyamide-elastane and polyester have a relatively narrow bandwidth of 178 and 130 MHz, respectively. We hypothesise that since the polyamide-elastane and polyester have a lower loss (loss tangent is between 0.005 and 0.015), the bandwidth of the antennas with substrates from these materials is more narrow versus the bandwidth of the antennas with denim and cotton substrates.

Figure 10b,c show the simulated radiation efficiency and maximum gain of the four antennas when they are in the free space. The behaviour of the radiation efficiency of the antennas, as a function of frequency, is shown in Figure 10b. From the results we can see that the radiation efficiency of the antennas with denim and cotton substrates remains almost unchanged (between 10% and 16%) across the MBAN and ISM bands. Furthermore, Figure 10b shows that the simulated radiation efficiency of the antenna with a polyamide-elastane substrate varies from 18% at 2.48 GHz to 30% at 2.42 GHz. The antenna with a substrate from polyester shows significant variations in the radiation efficiency from 28% at 2.36 GHz to 60% at 2.44 GHz.

The maximum gain of the antenna with a polyester substrate varies between −0.5 and 3.5 dBi across the MBAN and ISM bands, as seen in Figure 10c. The gain of the antenna with a polyamide-elastane substrate varies from −2.5 to 0 dBi. On the other hand, for antennas with cotton and denim substrates, small variations of only 1.5 dB in gain are observed. Also, the maximum gain of the antennas with denim and cotton substrates shows an increasing trend with increasing the frequency.

**Figure 11:**
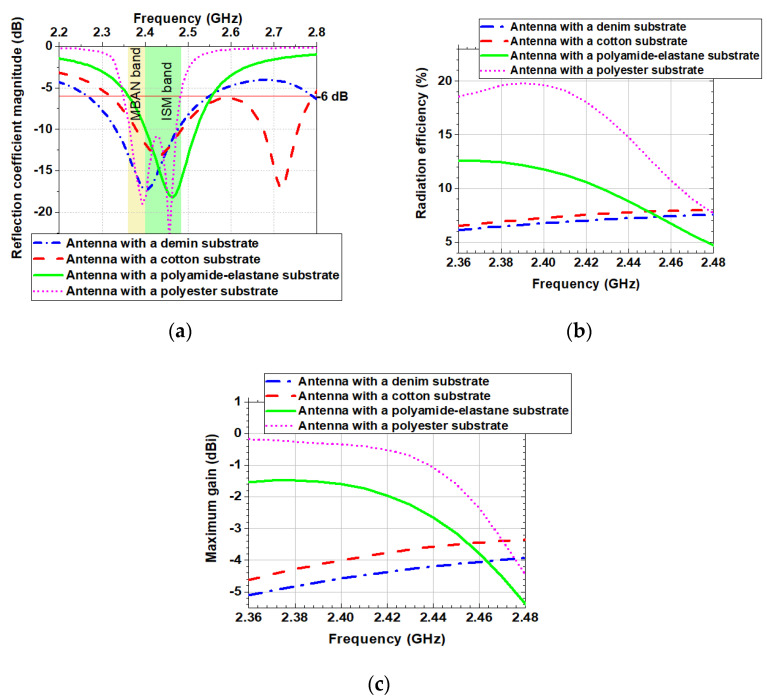
Simulated on the phantom: (**a**) Reflection coefficient magnitudes versus frequency; (**b**) Radiation efficiency versus frequency; (**c**) Maximum gain versus frequency.

The simulated reflection coefficient magnitudes when the antennas were placed on the human body model are shown in Figure 11a. A good impedance matching is maintained for all four antennas despite the slight change in reflection coefficient magnitudes. The results show that the resonant frequency of the antennas with a substrate from cotton and denim fabric is not affected when positioned on the phantom. Similar results are observed in [1]. The resonant frequency of the antennas with a substrate from polyester and polyamide-elastane is slightly shifted up. Moreover, when the antennas are placed on the phantom, their bandwidths remain unchanged compared to the free-space scenario. It can be concluded that the resonant frequency and bandwidth are insensitive to detuning when the textile antennas backed by a reflector are positioned on the phantom.

The investigations have shown that the phantom significantly affected both the maximum gain and radiation efficiency of the antennas. For the antennas with denim and cotton substrates, the maximum gain and radiation efficiency are decreased by a factor of about 0.5 compared to the free space scenario. The simulated radiation efficiency of the antenna with a polyamide-elastane substrate falls between 5% and 13%, while simulated radiation efficiency of the antenna with a polyester substrate falls between 8% and 20%. The observed reductions in radiation efficiency and maximum gain are due to the power absorbed at the substrate and human body model.

**Figure 12:**
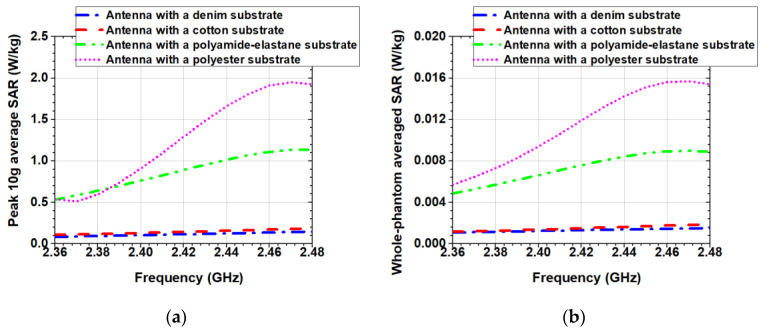
Simulated: (**a**) Peak 10 g average SAR (W/kg); (**b**) Whole-phantom averaged SAR (W/kg).

Figure 12a,b compare the computed peak 10 g average SAR and whole-phantom averaged SAR generated from each of the four antennas in the homogeneous phantom, in the MBAN and ISM bands. The results are normalized to a net input power of 100 mW. The antennas with denim and cotton substrates exhibit very low SAR values (below 0.02 W/kg), as shown in Figure 12a. On the other hand, 10 g average SAR of the antennas with polyamide-elastane and polyester substrates varies significantly from 0.5 to 1.15 W/kg and from 0.5 to 1.95 W/kg, respectively. From the results presented in Figure 12a it can be concluded that the peak 10 g average SAR for all four antennas is lower than that in the safety limit pointed in [24].

In MBAN and ISM bands, and for an input power of 100 mW, the whole-phantom averaged SAR for the antennas with denim and cotton substrates is below 0.002 W/kg (about 40 times lower than the maximum allowed value of 0.08 W/kg), as seen in Figure 12b. The obtained whole-phantom averaged SAR for the antennas with polyamide-elastane and polyester substrates is 10 and five times lower than the maximum allowed value of 0.08 W/kg, respectively.

Finally, the comparison between the antennas with substrates from natural fibres (cotton and denim) and substrates from synthetic fibres (polyamide-elastane and polyester) showed that the antennas with substrates from synthetic fibres give higher SAR values than the antennas with substrates from natural fibres (Figure 12).

Also, numerical simulations were performed for each antenna at distances 0 mm, 5 mm and 10 mm between the antenna and the human body model in order to investigate the effect of the EM properties of different textile materials on the robustness of the proposed designs to impact of the human body loading.

**Figure 13:**
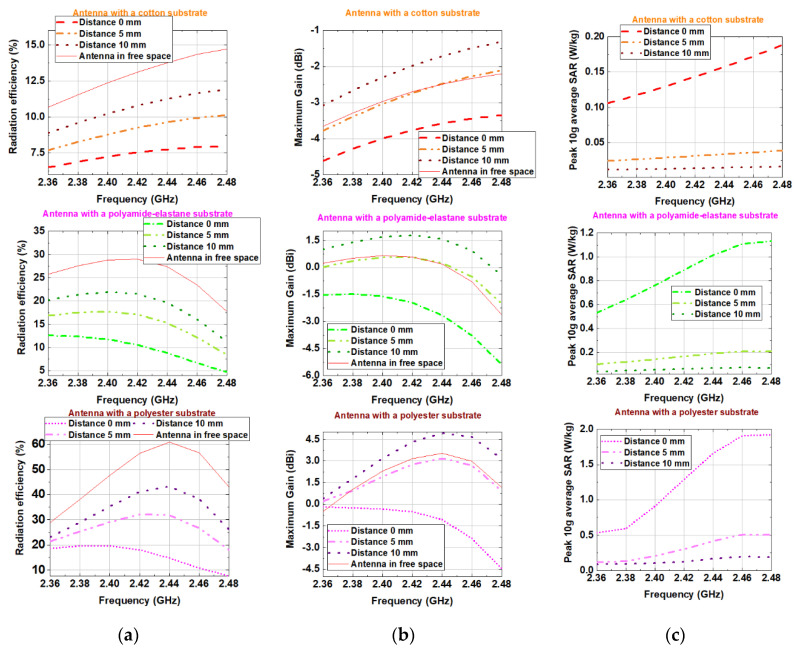
Simulated: (**a**) Radiation efficiency; (**b**) Maximum gain; (**c**) Peak 10 g average SAR versus frequency.

As might be expected, for each of the antennas the radiation efficiency and maximum gain increase with distance, while the peak 10 g average SAR decreases. From Figure 13b we also see that the gain at a distance 10 mm from the phantom for each antenna is larger than that in the free space. Consequently, we can conclude that the action of the phantom at a distance of 10 mm from the antenna is akin to a reflector, which enhances radiation in the direction opposed to the human body model.

Moreover, the computed maximum allowable net input powers which satisfy the ICNIRP restriction of 2 W/kg for peak 10 g average SAR for each antenna at different distances to the phantom are shown in Table 2. The maximum allowable net input power which satisfies the ICNIRP restriction at a distance of 0 mm is 1353 mW and 1062 mW for the antennas with denim and cotton substrates, respectively while for antennas with polyamide-elastane and polyester substrates is 176 mW and 104 mW, respectively.

**Figure 14:**
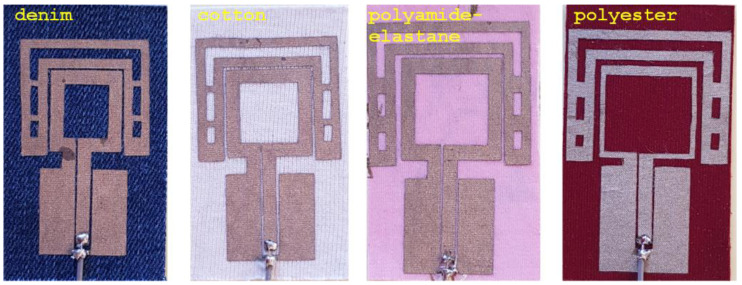
Photographs of the fabricated prototypes with a substrate from denim, cotton, polyamide-elastane and polyester.

Next, the prototypes of the antennas with cotton, polyamide-elastane and polyester substrates were fabricated using the procedure described in Section 2.3 and in [1]. Photographs of the fabricated prototypes are shown in Figure 14. The prototypes are very light (the weight of the prototype with a substrate from denim is 3.2 g, cotton 2.7 g, polyamide-elastane and polyester 2.6 g), which will allow the antennas to be easily integrated into clothing.

**Figure 15:**
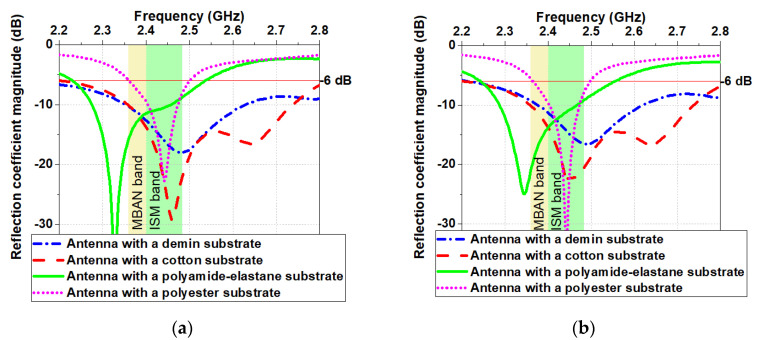
Measured reflection coefficient magnitudes when the antennas are: (**a**) in the free space; (**b**) on the homogeneous phantom.

The measured |S_11_| for all four prototypes are given in Figure 15. As can be seen, a good impedance matching is maintained in both scenarios (in free space and on the phantom). Also, a good agreement can be found between measured and simulated (see Figure 10a and Figure 11a) reflection coefficient magnitudes.

## 4. Conclusions

In this paper, we have presented a methodology for the design, fabrication and measurement of small wearable backed antennas for application in sensor nodes. Based on the presented design methodology, a new small-sized antenna with a denim substrate was proposed. Since the designed antenna is expected to be in close proximity to the human body during the real operating conditions in a W-WSN, the design and optimization were made both in the free space and on a human body model. In the free space, the antenna exhibits bandwidth (|S_11_|<−6 dB) of 285 MHz and radiation efficiency from 12% to 16% in MBAN and ISM bands. The simulated radiation efficiency varies from 6% to 7.8% when the antenna is placed on the phantom.

Concerning the health hazard, a more thorough evaluation and characterization of the SAR in the human body model were carried out. From the results, it can be concluded that the proposed antenna exhibits low SAR values (peak 10 g average SAR is about ten times lower than the maximum allowed value of 2 W/kg) due to the shielding effect of the reflector and also due to the unidirectional radiation pattern of the antenna.

The performance of the prototype of the proposed antenna was evaluated using passive and active tests. A good agreement between simulated and measured |S_11_| with a slight shift in the resonant frequency is observed. In order to get a complete performance of the fabricated prototype, range tests were performed in a semi-anechoic chamber and in a shielded room. Depending on the specific antenna position, the measured RSSIs vary between −44 and −69 dBm in the semi-anechoic chamber and between −37 and −56 dBm in the shielded room. Hence, it is possible to conclude that proposed antenna shows very good RSSIs which satisfy the requirement of the receiver sensitivity in W-WSNs (−94 dBm).

To investigate the impact of the textile materials on the antenna performance, the antenna geometry was studied on four textile substrates (denim, cotton, polyamide-elastane and polyester). The reflection coefficient magnitudes, bandwidth, maximum gain and radiation efficiency of the four antennas were numerically studied and compared to two scenarios: (1) in the free space and (2) on the semisolid phantom. The numerical investigations reveal that in MBAN and ISM bands, each of these four textile antennas achieves stable performance in both scenarios.

From the results, we can conclude, that the real part of the permittivity of the antenna substrate has consequences on the overall antenna size and resonant frequency. As expected, with increasing the substrate material permittivity, the overall size of the antenna decreases. Results show that the gain and radiation efficiency decrease with increasing dielectric losses in the textile substrate. Results for peak 10 g average SAR have revealed that the antennas with denim and cotton substrates exhibit SAR values below 0.02 W/kg. On the other hand, the peak 10 g average SAR of the antennas with polyamide-elastane and polyester substrates varies significantly from 0.5 to 1.5 W/kg and from 0.5 to 1.95 W/kg, in the frequency range of 2.36 to 2.48 GHz. Consequently, the peak 10 g average SARs are sensitive to substrate material dielectric loss.

In order to verify the results from the numerical simulations, prototypes of the antennas were fabricated, and their parameters were measured in a semi-anechoic chamber. A good agreement is found between measured and simulated reflection coefficient magnitudes of the antenna prototypes for two scenarios when the antennas are: (1) in the free space and (2) on the semisolid phantom. Therefore, the proposed small textile antennas backed by a reflector are promising candidates for integration into garments for applications in W-WSNs.

## Figures and Tables

**Table 1:** Target antenna parameters and characteristics.

Antenna Specification	
Frequency range (GHz)	2.36–2.48
|S_11_| in operating frequency range (dB)	≤−6
Minimum radiation efficiency on the human body (%)	≥5
Radiation pattern	unidirectional
Minimum front to back ratio (dB)	≥10
Peak 10 g average SAR (W/kg)	≤2
Maximum antenna size (cm^2^)	≤25
Maximum antenna profile (mm)	≤1.5
Maximum antenna weight (g)	≤5

**Table 2:** Maximum allowable net input power which satisfies the ICNIRP restriction of 2 W/kg for peak 10 g average SAR in the homogeneous phantom at 2.48 GHz.

Distance, mm	Maximum Net Input Power, mW			
	Denim	Cotton	Polyamide-Elastane	Polyester
0	1353	1062	176	104
5	4818	5133	961	397
10	9833	12,070	2806	1065

## References

[1] Atnasova G.L., Atanasov N.T. Impact of electromagnetic properties of textile materials on performance of a low-profile wearable antenna backed by a reflector. Proceedings of the 2020 International Workshop on Antenna Technology (iWAT).

[2] Atanasov N.T., Atanasova G.L., Atanasov B.N., Al-Rizzo H. (2020). Wearable Textile Antennas with High Body-Area Isolation: Design, Fabrication, and Characterization Aspects. Modern Printed Circuit Antennas.

[3] Antolin D., Medrano N., Calvo B., Pérez F. (2017). A wearable wireless sensor network for indoor smart environment monitoring in safety applications. Sensors.

[4] Lin R., Kim H.-J., Achavananthadith S., Kurt S.A., Tan S.C.C., Yao H., Tee B.C.K., Lee J.K.W., Ho J.S. (2020). Wireless battery-free body sensor networks using near-field-enabled clothing. Nat. Commun..

[5] Al-Sehemi A.G., Al-Ghamdi A.A., Dishovsky N.T., Atanasov N.T., Atanasova G.L. (2018). Design and performance analysis of dual-band wearable compact low-profile antenna for body-centric wireless communication. Int. J. Microw. Wirel. Technol..

[6] El Attaoui A., Hazmi M., Jilbab A., Bourouhou A. (2019). Wearable wireless sensors network for ECG telemonitoring using neural network for features extraction. Wirel. Presonal Commun..

[7] Seimeni M.A., Tsolis A., Alexsandridis A.A., Pantelopoulos S.A. The effects of ground-plane of a textile higher mode microstrip patch antenna on SAR. Proceedings of the 2020 International Workshop on Antenna Technology (iWAT).

[8] Al-Sehemi A.G., Al-Ghamdi A.A., Dishovsky N.T., Atanasov N.T., Atanasova G.L. (2017). Flexible and small wearable antenna for wireless body area network applications. J. Electromagn. Waves Appl..

[9] Song Y., Le Goff D., Riondet G., Mouthaan K. Polymer-based 4.2 GHz patch antenna. Proceedings of the 2020 International Workshop on Antenna Technology.

[10] Ashyap A.Y.I., Dahlan S.H., Abidin Z.Z., Dahri M.H., Majid M.K.A.R.H.A., Kamarudin M.R., Yee S.K., Jamaluddin M.H., Alomainy A., Abbasi Q.H. (2020). Robust and efficient integrated antenna With EBG-DGS enabled wide bandwidth for wearable medical device applications. IEEE Access.

[11] Ashyap A.Y.I., Marzudi W.N.N.W., Abidin Z.Z., Dahlan S.H., Majid H.A., Kamaruddin M.R. Antenna incorporated with Electromagnetic Bandgap (EBG) for wearable application at 2.4 GHz wireless bands. Proceedings of the 2016 IEEE Asian-Pacific Conference on Applied Electromagnetics.

[12] Ashyap A.Y.I., Abidin Z.Z., Dahlan S.H., Majid H.A., Shah S.M., Alomainy A. (2017). Compact and low-profile textile EBG-based antenna for wearable medical applications. IEEE Antennas Wirel. Propag. Lett..

[13] Del-Rio-Ruiz R., Lopez-Garde J.M., Legarda J. (2019). Planar taxtile off-body communication antennas: A survey. Electronics.

[14] Kapse S., Gundre S.B. Fractal shaped dual-band EBG integrated textile antenna. Proceedings of the 2019 4th International Conference on Recent Trends on Electronics, Information, Communication & Technology (RTEICT-2019).

[15] Atanasov N.T., Atanasova G.L., Stefanov A.K., Nedialkov I.I. (2019). A wearable, low-profile, fractal monopole antenna with a reflector for enhancing antenna performance and sar reduction. Proceedings of the 2019 IEEE MTT-S International Microwave Workshop Series on Advanced Material and Processes for RF and THz Applications (IMWS-AMP).

[16] Alemaryeen A., Noghanian S. Performance analysis of textile AMC antenna on body model. Proceedings of the 2017 United States National Conference of URSI National Radio Science Meeting (USNC-URSI NRSM).

[17] Alemaryeen A., Noghanian S. (2019). On-body low-profile textile antenna with artificial magnetic conductor. IEEE Trans. Antennas Propag..

[18] Fang R., Song R., Zhao X., Wang Z., Qian W., He D. (2020). Compact and loa-profile UWB antenna based on graphene-assembled films for wearable applications. Sensors.

[19] Androne A., Tamas R.D., Tasu S. Influence of the substrate material on the radar cross section of square loop unit cells for frequency selective surfaces. Proceedings of the 2020 International Workshop on Antenna Technology (iWAT).

[20] Zhu J., Cheng H. (2018). Recent development of flexible and stretchable antennas for bio-integrated electronics. Sensors.

[21] Nepa P., Rogier H. (2015). Wearable antennas for off-body links at VHF and UHF bands: Challenges, the state of art, and future trends below 1 GHz. IEEE Antennas Propag. Mag..

[22] Jiang Z.H., Brocker D.E., Sieber P.E., Werner D.H. (2014). A Compact, low-profile metasurface-enabled antenna for wearable medical body-area network devices. IEEE Trans. Antennas Propag..

[23] Rowell C., Lam E.Y. (2012). Mobile-phone antenna design. IEEE Antennas Propag. Mag..

[24] ICNIRP (1998). Guidelines for limiting exposure to time-varying electric, magnetic, and electromagnetic fields (up to 300 GHz). Health Phys..

[25] IEEE Standards Coordinating Committee (2006). IEEE for Safety Levels with Respect to Human Exposure to Radio Frequency Electromagnetic Fields, 3 kHz to 300 GHz.

[26] Al-Naiemy Y., Elwi T.A., Khaleel H.R., Al-Rizzo H. (2012). A systematic approach for the design, fabrication, and testing of microstrip antennas using inkjet printing technology. ISRN Commun. Netw..

[27] Zhong J., Kiourti A., Sebastian T., Bayram Y., Volakis J.L. (2016). Conformal load-bearing spiral antenna on conductive textile threads. IEEE Antennas Wirel. Propag. Lett..

[28] Balanis C.A. (1996). Antenna Theory Analysis and Design.

[29] Arai H. (2013). Measurement of Mobile Antenna Systems.

[30] Chen Z.N. (2007). Antennas for Portable Devices.

[31] Yilmaz T., Foster R., Hao Y. (2014). Broadband tissue mimicking phantoms and a patch resonator for evaluating noninvasive monitoring of blood glucose levels. IEEE Trans. Antennas Propag..

[32] Lam P.T., Le T.Q., Le N.N., Nguyen S.D. (2017). Wireless sensing modules for rural monitoring and precision agriculture applications. J. Telecommun. Inf. Technol..

